# Stem elongation and gibberellin response to submergence depth in clonal plant *Alternanthera philoxeroides*

**DOI:** 10.3389/fpls.2024.1348080

**Published:** 2024-05-24

**Authors:** Shufang Jing, Xinyi Ren, Feng Lin, Hangang Niu, Qiaoli Ayi, Binna Wan, Bo Zeng, Xiaoping Zhang

**Affiliations:** ^1^ Key Laboratory of Eco-environments in Three Gorges Reservoir Region (Ministry of Education), Chongqing Key Laboratory of Plant Ecology and Resources in Three Gorges Reservoir Region, School of Life Sciences, Southwest University, Chongqing, China; ^2^ School of Biological Science and Food Engineering, Huanghuai University, Zhumadian, China

**Keywords:** clonal plant, alligator weed, gibberellin, hydrostatic pressure, submergence times, submergence depth

## Abstract

Clonal plants are widely distributed in the riparian zone and play a very important role in the maintenance of wetland ecosystem function. Flooding is an environmental stress for plants in the riparian zone, and the response of plants varies according to the depth and duration of flooding. However, there is a lack of research on the growth response of clonal plants during flooding, and the endogenous hormone response mechanism of clonal plants is still unclear. In the present study, *Alternanthera philoxeroides*, a clonal plant in the riparian zone, was used to investigate the time-dependent stem elongation, the elongation of different part of the immature internodes, and the relationship between growth elongation and the phytohormone gibberellin (GA) under a series of submergence depths (0 m, 2 m, 5 m, and 9 m). The results showed that stem elongation occurred under all treatments, however, compared to 0 m (control), plants grew more under 2 m and 5 m submergence depth, while grew less under 9 m water depth. Additionally, basal part elongation of the immature internode was the predominant factor contributing to the stem growth of *A. philoxeroides* under different submergence depths. The phytohormone contents in basal part of the mature and immature internodes showed that GA induced the differential elongation of internode. Plant submerged at depth of 2 m had the highest GA accumulation, but plant submerged at depth of 9 m had the lowest GA concentration. These data suggested that GA biosynthesis are essential for stem elongation in *A. philoxeroides, and* the basal part of the immature internode was the main position of the GA biosynthesis. This study provided new information about the rapid growth and invasion of the clonal plant *A. philoxeroides* around the world, further clarified the effects of submergence depth and duration on the elongation of the stem, and deepened our understanding of the growth response of terrestrial plants in deeply flooded environments.

## 1 Introduction

Flooding is an environmental stress for plants in the riparian zone, and the response of plants varies according to the depth and duration of flooding (Müller et al., 2019). Flooding depth and duration concomitantly influence the growth traits and yield of plant (Meng et al., 2022). A longer waterlogging duration caused a greater reduction in the above parameters (Wang et al., 2017). Clonal plants are widely distributed in the riparian zone and play a very important role in the maintenance of wetland ecosystem function, and often adapt to environmental changes through phenotypic plasticity, especially invasive clonal plants (Dong et al., 2017; Guo et al., 2023). Previous studies have demonstrated that the clonal plant *Alternanthera philoxeroides* is an invasive amphibious weed that is native to South America but has now invaded into the temperate and tropical regions across the world (Wu et al., 2017). *A. philoxeroides* has rapid clonal reproduction and is phenotypically plastic (leaf area, internode length, shoot diameter, etc.) (Gao et al., 2015).

Compared to the terrestrial environmental factors, the environmental factors in the water body can change considerably, such as light, water temperature, dissolved O_2_ and CO_2_ concentrations, etc (Vervuren et al., 2003). Light quantity and quality in rivers vary with depth and turbidity, for example, in the River Rhine, light quantum flux decreases with increasing water depth both in freshwater lakes and in flooded environments (Vervuren et al., 1999; Li et al., 2024). Light is reduced to 90% of the total solar radiation entering the water column at a depth of 50 cm, and less than 1% of the light intensity is available underwater when the water depth reaches more than 1.5 m (Vervuren et al., 2003). In completely submerged environments, O_2_ concentration in plants is reduced and low levels of O_2_ stimulate ethylene biosynthesis, whose diffusion rate in water is very slow, leading to a rapid rise in ethylene levels in plants in a short period of time (Voesenek et al., 2016; Wang and Komatsu, 2022).

The clonal plant *A. philoxeroides* is a common plant in floodplains, riparian zones, and water-level drawdown zones of large reservoirs with inundation-disturbed habitats (Yang et al., 2019), and it is also distributed in areas with deeper inundation (Zheng et al., 2021). For example, the Three Gorges Reservoir (TGR) which is the largest hydroelectric power project in the world, the water level of the reservoir fluctuates regularly from 145 m to 175 m in elevation (Chen et al., 2021). Thus, the water-level drawdown zones with a maximum drop of 30 m are formed along the banks of the Yangtze River (Lei et al., 2017). We have been conducting long-term research on plant growth in the drawdown zone, and we have found that the clonal plant *A. philoxeroides* exhibits a fast-growing in shallow submerged environments and a slow-growing or even stop-growing in deep submerged conditions (Ayi et al., 2016; Jing et al., 2022), but there is little discussion on the growth response of *A. philoxeroides* at different water depths and its response mechanism. Our previous study has shown that the formation of pith cavity and adventitious roots, and non-structural carbohydrate metabolism play important roles in the changes of different growth strategies in *A. philoxeroides* (Jing et al., 2022). The stem growth of *A. philoxeroides* at any submergence depth was chiefly caused by the elongation of the basal parts of immature internodes, which was highly correlated to both cell proliferation and cell enlargement (Jing et al., 2024). However, the time depended stem elongation and the hormone regulatory mechanisms remain unclear, this characteristic of the plant species is crucial for explaining the successful invasion of clonal plants (You et al., 2016).

Phytohormone plays a very important role as a signalling substance in the response of plants to biotic and abiotic stresses (Taiz and Zeiger, 2010). The response and adaptation strategies of plants in flooded environments are very closely related to hormone concentrations, and the regulation of plant morphology, anatomy, physiology, ecology, molecules and signalling under low oxygen or low light stress conditions is largely influenced by hormones (Bailey-Serres and Voesenek, 2008; Lin et al., 2021). When plants were submerged, the content of ethylene increases due to the diffusion of ethylene is weakened, which inhibits abscisic acid (ABA) synthesis and promotes ABA decomposition, whereas ABA inhibits gibberellin (GA) synthesis (Fukao and Bailey-Serres, 2008), so an increase in ethylene promotes an increase in GA content, which in turn promotes stem growth (Sasidharan and Voesenek, 2015). Physiological and genetic analyses indicated that GA biosynthesis and signal transduction are essential for internode elongation in deep-water rice (Ayano et al., 2014). Its role in plant response to submergence has been widely reported (Sasidharan and Voesenek, 2015; Wang et al., 2021). However, changes in the concentration of the endogenous hormone GA in different water depth environments have not been reported for the clonal plant *A. philoxeroides.*

To explore the time-dependent stem elongation and the hormone regulatory mechanisms under different submergence depths, taking the clonal plant *Alternanthera philoxeroides* (Mart.) Griseb., a submergence-tolerant plant as a model, we hope to solve the following scientific questions in this study: (1) What are the trends in the stems of different maturity levels of *A. philoxeroides* as the duration of submergence changes? (2) Is there a difference in the content of endogenous hormone GA in the immature internodes of *A. philoxeroides* under different water-depths? In order to clarify the above questions, we measured the length of stem, immature stem of *A. philoxeroides* at different water-depths, and determined the endogenous hormone GA content of mature and immature stem by using high-performance liquid chromatography and quantified by tandem mass spectrometry (HPLC-MS/MS). The answers to these questions will help understanding the phytohormone regulatory mechanisms of clonal plant tolerance to extreme flooding and explain why *A. philoxeroides* remains highly invasive worldwide.

## 2 Materials and methods

### 2.1 Plant material and cultivation

*Alternanthera philoxeroides* (Mart.) Griseb. can spread quickly via clonal growth, in this study, plants were grown as described in Jing et al. (2022). *A. philoxeroides* plants were cultivated from cuttings obtained from plants naturally growing on the banks of the Jialing River in Chongqing, Southwest China (29^○^49’42’’N, 106^○^26’46’’E). Each selected cutting was planted in a plastic pot (diameter and depth were both 13 cm) containing riparian soil from the Jialing River banks. All plants were cultivated under the same conditions. The temperature, relative humidity, daily maximum light (PAR) intensity, and water provision were maintained at 10~15 °C, 75~85%, 600~800 µmol m^–2^ s^–1^, and approximately 80~90% of the soil water-holding capacity, respectively. After approximately one month of cultivation, plants with approx. 288 mm height and 12 internodes were selected for submergence treatments.

### 2.2 Experimental design

The plants subjected to complete submergence treatments were suspended at planned water depths, as described in Jing et al. (2022). The design of 2 m, 5 m, and 9 m deep submergence was based on our long-term field observation on the elevational distribution of *A. philoxeroides* in the water level fluctuation zone of the Three Gorges reservoir. Unsubmerged control plants were placed under dark conditions and watered regularly to ensure adequate water supply. Four submergence treatments were applied in a fully randomized design using selected plants. Control plants were placed under dark conditions and were not submerged, in this article it is called 0 m. Additionally, three groups of plants were submerged in a water-filled concrete reservoir, with the top of plants 2 m, 5 m, and 9 m beneath the water surface (**Supplementary Figure S1A**). The plants in pots were suspended at planned water depths as described in Jing et al. (2022). According to the previous observation and pre-experimental results, the submergence treatments lasted to 11 d in the growth measured experiments, but 4 d in the endogenous GA measurements.

To investigate the effects of submergence depth on plants, the physicochemical status of water body (light, dissolved oxygen (DO), pH, and temperature) in the concrete reservoir were kept constant at any depths as described in Jing et al. (2022). DO concentration, photosynthetically active radiation (PAR) intensity, temperature and pH of the water column at different depths in the reservoir were checked twice per day (morning and evening) using a multi-parameter water quality analyzer (Hydrolab DS5, Hach, United States). During the experiment, no significant differences in these factors were found between different water depths (**Table 1**).

**Table 1:** Physico-chemical properties of water body in submergence reservoir during the experiment.

Submergence Depth (m)	Dissolved oxygen concentration (mg L^-1^)	Temperature (°C)	pH	PAR (μmol m^-2^ s^-1^)
0	n.a.	25.46 ± 0.03 a	n.a.	0 a
2	8.25 ± 0.08 a	25.48 ± 0.06 a	7.02 ± 0.02 a	0 a
5	8.16 ± 0.07 a	25.45 ± 0.07 a	7.01 ± 0.02 a	0 a
9	8.11 ± 0.06 a	25.46 ± 0.04 a	7.03 ± 0.01 a	0 a

The dissolved oxygen, temperature, photosynthetically active radiation (PAR), and pH of the water body in the concrete reservoir for submergence treatments were checked at different depths twice per day (in the morning and evening) using a multi-parameter water quality analyzer (Hydrolab DS5, Hach, United States) during the experiments (mean ± s. e.; n = 20); n.a. indicates no data. Same lower-case letter indicates no significant difference (p > 0.05) between submergence depths.

### 2.3 Growth measurements

Each plant had approximately 12 internodes at the start of treatments. From the stem base upwards, the 1st to 6th internodes were relatively more mature and the 7th to 12th internodes were immature (**Supplementary Figure S1B**). We marked nondestructively immature stems so as to distinguish the mature, immature stems formed before treatment. The length of mature stems, immature stems were measured every day. As plants may produce gaseous substances such as ethylene during submergence, it was ensured that the measurements were taken underwater, at the same time, the plants are not exposed to the atmosphere. Once the daily measurement was completed, the plants were quickly submerged to the appropriate water-depth for continuation of the treatment. In order to investigate the submergence time-dependent growth pattern of different parts of immature internodes, we selected an immature internode (the length of the internode was usually between 2.5 and 3.2 cm, as shown in **Supplementary Figure S1D**) from each plant before treatments and divided the internode into three equilong parts (basal, middle, and upper part, as shown in **Supplementary Figure S1E**) by marking with red polyester threads (Jing et al., 2024), measured the lengths of all parts every day.

### 2.4 Phytohormone concentration analysis

The endogenous GA concentration in the basal part of the internode of *A. philoxeroides* plants were mainly composed of GA_1_, GA_3_, GA_4_, and GA_7_. According to the results of the pre-experiment and the research objectives of the present study, the growth of plants under different submergence depths had already shown significant differences at the 4th day, and the endogenous hormone content of the plants was relatively high, the sampling time was set as the 4th day. The basal parts of each internode were harvested after submergence or control treatments, frozen in liquid nitrogen immediately, and kept at -80 °C before freeze drying. Ten basal parts of the mature and immature internodes were pooled to obtain enough material per sample. There were three replicates for GAs (GA_1_, GA_3_, GA_4_ and GA_7_) analyses. Measurement of endogenous GAs concentration was performed by HPLC-MS/MS. The HPLC-MS/MS system was composed of a high-performance liquid chromatography (HPLC, Agilent Technologies 1200 series, USA) connected to AB Sciex API 6500 Qtrap mass spectrometer (Concord, ON, Canada). The Analyst 1.6.3 software (Concord, ON, Canada) controlled the HPLC-MS/MS system and Multiquant 3.2 to process the data (Pan et al., 2010).

### 2.5 Statistical analysis

Elongation difference of stem (or immature stem) between submergence depths were checked by one-way ANOVA. Separate ANOVA with Repeated Measures was used to detect the difference in stem length, basal, middle, and upper parts within internodes, One-way ANOVA was used to examine the difference in contents of gibberellin in mature and young stems respectively between different treatments. Logarithm data transformation was performed to equalize variance if necessary. Differences between treatments were detected using the Tukey HSD test, and the significance level was set at *p* = 0.05. All analyses were conducted using SPSS 22 (SPSS Inc., Chicago).

## 3 Results

### 3.1 Elongation of stem and immature stem parts

**Figure 1:**
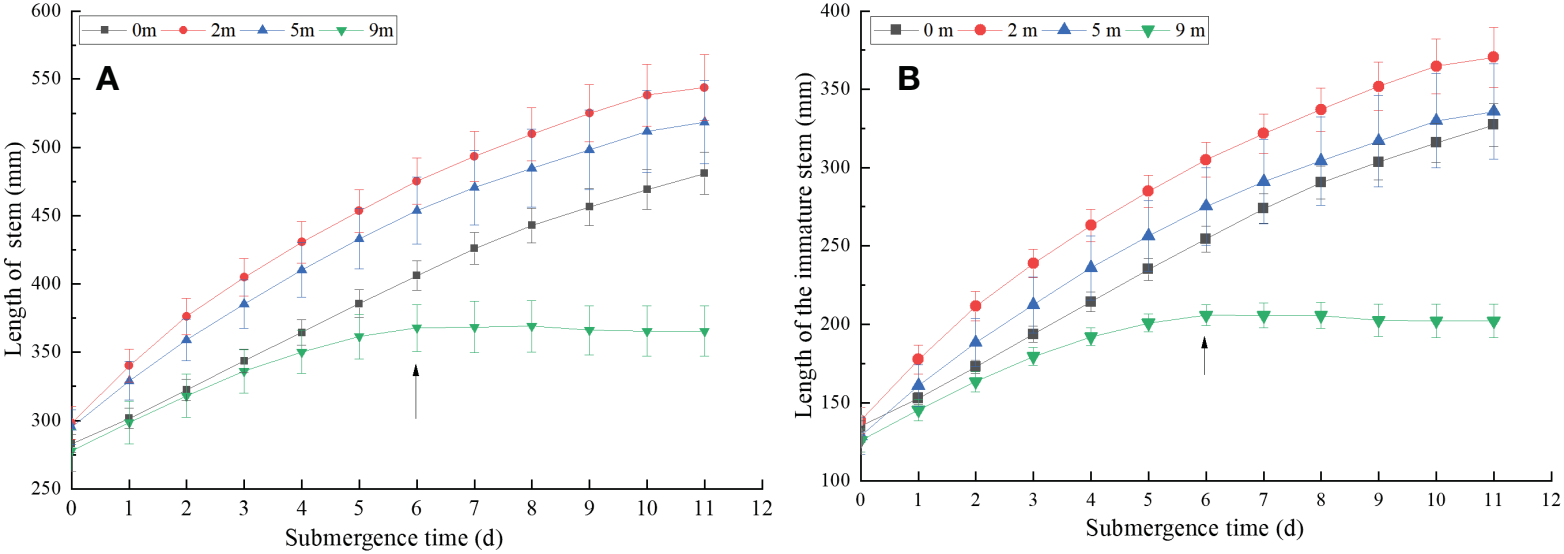
Length of stem **(A)** and immature stem **(B)** of *Alternanthera philoxeroides* after the start of different submergence treatments (Mean ± s. e.; n = 6). The arrows indicate *A philoxeroides* stops growing at 9 m water depth.

*A. philoxeroides* plants subjected to four treatments all elongated their stems during the experiment day by day (**Figure 1A**). At the end of treatments, the stem elongation were 198.00 mm, 245.67 mm, 223.17 mm, and 87.50 mm averagely in plants submerged at water depths of 0 m (control), 2 m, 5 m, and 9 m, respectively (**Figure 1A**). Compared to the 0 m, the stem presented apparent elongation when submerged at water depth of 2 m and 5 m, but the stem only had very slight elongation when submerged at water depth of 9 m. Stem elongation decreased with increasing submergence depth after 6 days treatment (**Figure 1A**). From the 7th day, the stem elongation of *A. philoxeroides* was significantly inhibited in the 9 m submergence depth, no elongation was observed. At this time, the stem elongation of plants submerged at water depth of 2 m and 5 m were still faster, indicating that the growth response of the stem of *A. philoxeroides* varied greatly at different water depths (**Figure 1A**). It was shown that water depth of 2 m and 5 m promoted elongation of the stems, while water depth of 9 m inhibited elongation of the stems, and this inhibition was more severe from the 7th day after the onset of submergence.

The immature stem elongation trend (**Figure 1B**, the 7th to 12th internodes) of *A. philoxeroides* under different water depths was consistent with that of the stems (**Figure 1A**). At the end of treatments, the elongation of immature stems in plants submerged at water depth of 0 m (control), 2 m and 5 m were 192.17 mm, 231.67 mm, and 206.83 mm, respectively (**Figure 1B**), and the immature stems were rapidly elongated throughout the treatment period. However, the water depth of 9 m promoted plant growth during the first 6 days of the experiment and then turned to inhibit plant growth as the duration of submergence increased (**Figure 1B**, especially on 7th day after the start of submergence), during the whole treatment period, the immature stem only grew 76.33 mm averagely.

### 3.2 Elongation of the different parts of immature internodes

**Figure 2:**
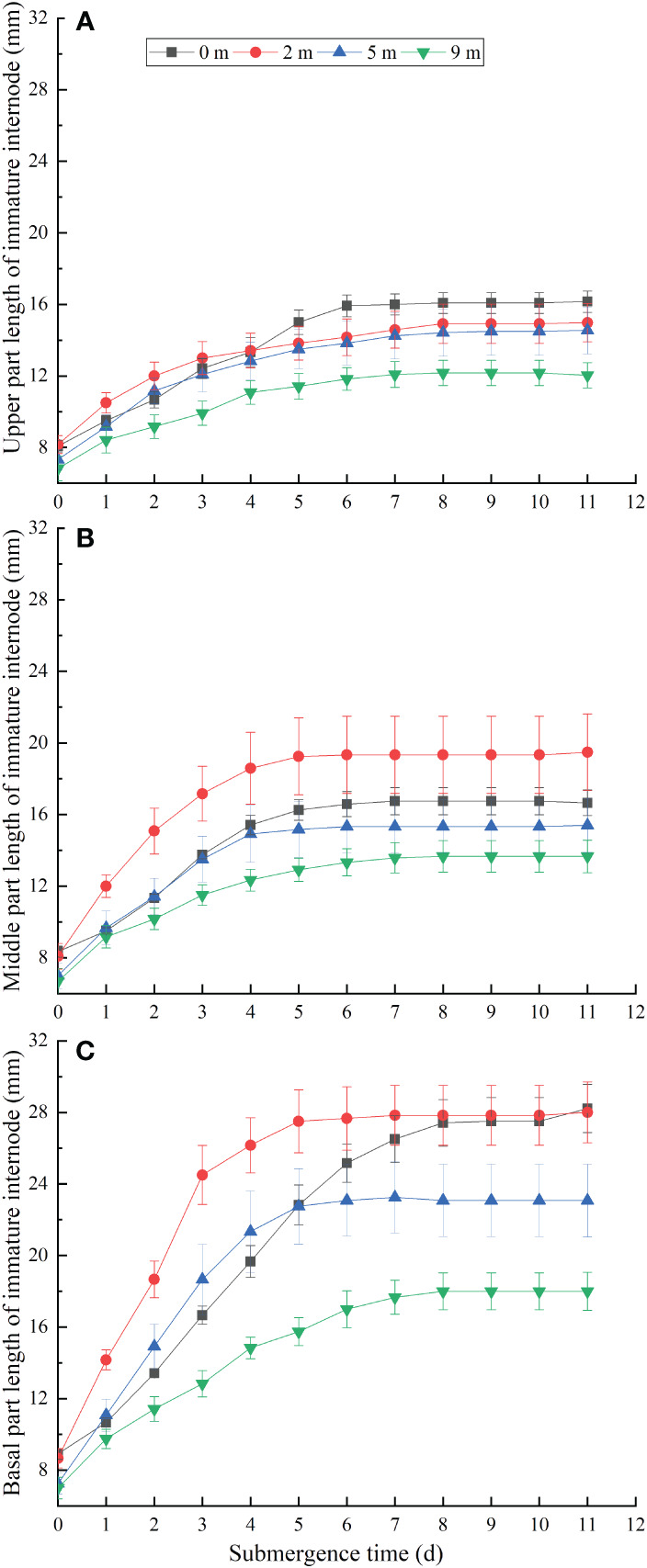
Upper **(A)**, middle **(B)** and basal **(C)** length of the immature internode for *Alternanthera philoxeroides* after the start of different submergence treatments (Mean ± s. e.; n = 6).

The elongation of the basal (**Figure 2A**), middle (**Figure 2B**) and upper (**Figure 2C**) of immature internodes of *A. philoxeroides* showed different growth trends, which grew from about 8 mm at the start of the treatments to about 19 mm, 11 mm and 6 mm at 7 day under 2 m water depth, respectively. The growth rate of the basal parts was significantly faster than that of the middle parts, which in turn was faster than that of the upper parts (**Figures 2A–C**). However, when submergence treatment was carried out for 8 days, there was no significant growth in all parts of the marked immature internode, except for the internodes of the 0 m (unsubmerged group). In the same period of time, the elongation of basal, middle, and upper parts gradually decreased with increasing water depth (**Figures 2A–C**). Overall, 2 m water depth promoted the basal parts elongation of immature internodes at the early stage of treatments, while 9 m water depth had a certain inhibitory effect on the elongation of the immature internodes, and 5 m water depth had faster growth than 0 m at the beginning of submergence for 1~4 days, but the final length of all parts of the immature internodes was lower than that of the 0 m (**Figure 2C**).

### 3.3 Endogenous GA concentration in the basal part of the internode

**Figure 3:**
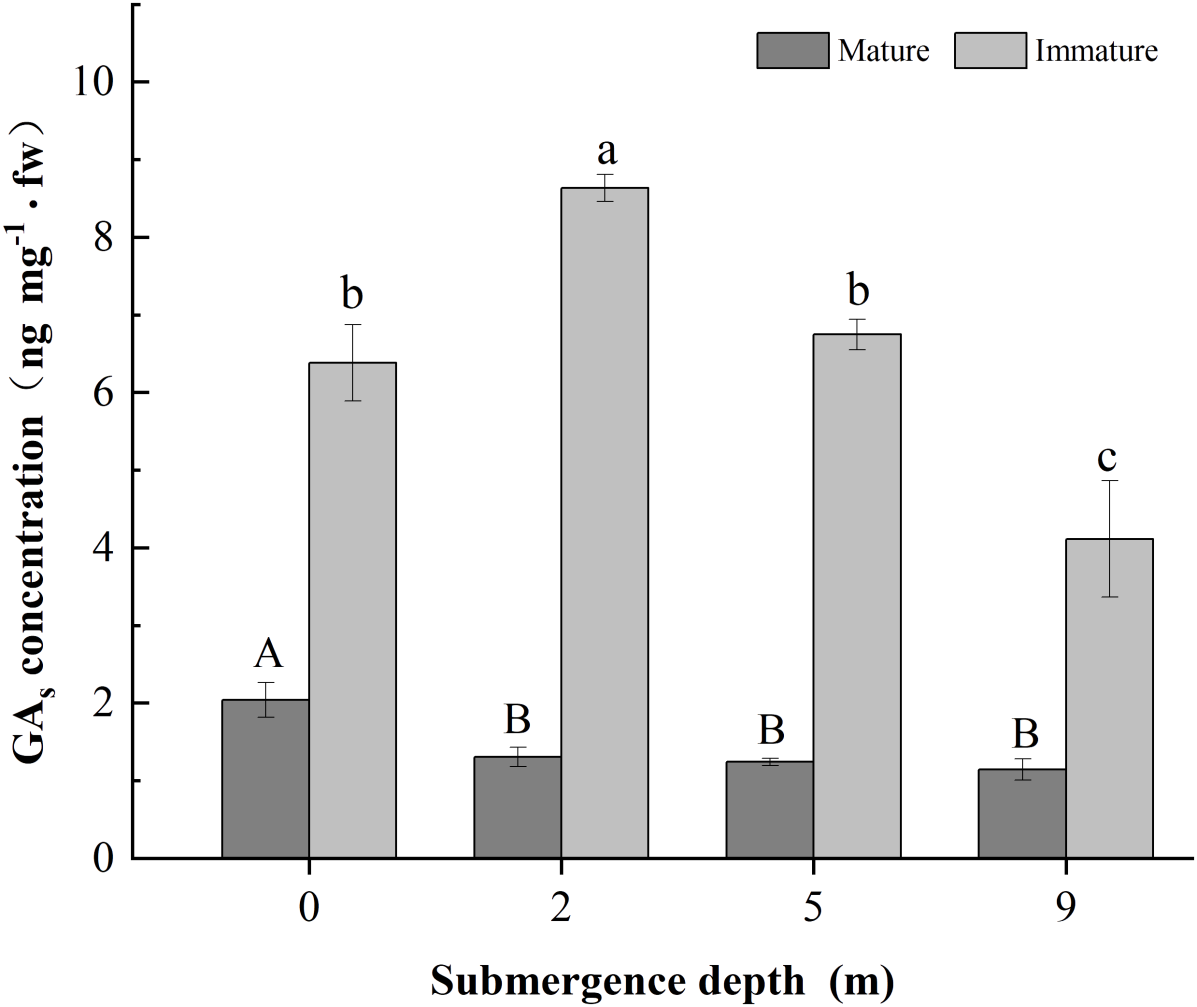
Concentration of GA_S_ in the basal of mature and immature internodes of *Alternanthera philoxeroides* at the fourth day of the submergence treatments (Mean ± s. e.; n =3). Different upper-case letters indicate statistically significant differences (*p* < 0.05) between mature internode for treatments. Different lower-case letters indicate statistically significant differences (*p* < 0.05) between mature stems for treatments. fw indicates fresh weight.

The concentration of GAs in immature internodes was higher than that in the mature internodes of *A. philoxeroides* (**Figure 3**). The concentration of GAs in the basal parts of immature internodes decreased with increasing submergence depth at the fourth day of experiment (*p* < 0.05, **Figure 3**). Plants submerged at 2 m water depth had the highest GAs concentration but plants submerged at 9 m depth had the lowest GAs concentration (*p* < 0.05, **Figure 3**). However, no significant difference was found between 0 m and 5 m submergence depths in GAs concentration in immature stems of *A. philoxeroides* (*p* > 0.05, **Figure 3**). The concentration of GAs in mature internodes of unsubmerged plants was significantly higher than that of submerged plants, but no difference was found among the three submerged treatments (i.e., 2 m, 5 m and 9 m). This indicates that 2 m water depth promoted GAs biosynthesis in immature internodes, whereas 9 m water depth inhibited GAs biosynthesis.

## 4 Discussion

The influences of submergence on plant growth are different according to different flooding depths and durations. It has been reported that the yield of rice is almost non-existent when the flooding time is 5~6 days (Meng et al., 2022), longer flooding durations limited the basal area growth of larger trees and reduced sexual reproduction (Greet et al., 2020). In the present study, the stems of *A. philoxeroides* were elongating with the durations of submergence in all treatments (**Figures 1A, B**), this is mainly due to the growth of immature internodes. But the elongation of immature stems decreased when water depth increased (**Figures 1A, B**). This is consistent with the results of our previous studies, which found that immature internodes comparatively made the largest contribution to plant stem elongation (Jing et al., 2022). Moreover, as previous studies have found that the immature internodes showed intra-internodal variation in elongation among their basal, middle, and upper parts, and the variation was affected by submergence depth (Jing et al., 2024). The basal parts achieved much longer elongation than the middle and upper parts at 2 m water depth, but this elongation difference faded away when the water depth increased gradually to 9 m (**Figures 2A–C**).

In fact, after 7 days of submergence, the stems of *A. philoxeroides* were no longer growing under 9 m water depth (**Figure 1A**), the same to the immature stems (**Figure 1B**). Plants submerged at depth of 0 m, 2 m, 5 m continued to grow until the experiment was terminated after 11 days of submergence (**Figures 1A, B**). However, elongation of immature internodes was over by day 5 (**Figure 2A–C**), suggesting that *A. philoxeroides* produces new internodes to increase its total stem length during submergence. This is consistent with our previous findings that *A. philoxeroides* can produce new internodes during submergence, but very few new internodes were produced during submergence at water depth of 9 m, and 1.85, 2.40, 1.85 new internodes on average were produced at water depth of 0 m, 2 m, and 5 m, respectively (Jing et al., 2022). This is an important feature for clonal plants to be able to reproduce in stressful environments (Jing et al., 2022).

It has been shown that ethylene content in plants rises rapidly within a short period of time in a completely submerged environment (Raskin and Kende, 1984; Voesenek et al., 2013, Voesenek et al., 2016), and that ethylene markedly increases the activity of the endogenous hormone GA, which in turn promotes cell division and cell elongation, and ultimately plant growth (Sasidharan and Voesenek, 2015). Therefore, in this study, when *A. philoxeroides* was completely submerged in a 2 m water depth environment, as in the completely submerged environment, the stem length was increased due to the rapid increase of ethylene concentration thereby inducing an increase in the biosynthesis of GAs, which promotes elongation and growth of the plant’s immature parts (**Figure 3**). GA promotes cell division and cell elongation (Sauter and Kende, 1992), this is consistent with our previous study, the difference in the internode elongation is mainly due to the difference in cell growth and development. Cells in the basal parts of immature internodes were shorter and numerous, whereas those in the middle and upper parts were relatively longer and smaller in number (Jing et al., 2024). Plants possess higher concentrations of GA under 2 m water depth, but have lower concentrations of growth-promoting GA and less plant growth under 9 m water depth (**Figure 3**). In our experimental system, all factors except water depth were kept constant (**Table 1**). The main difference between 2 m and 9 m of complete submergence is the difference in hydrostatic pressure. For every 1 m increase in water depth, the pressure of water acting on an object increases by about 9.8 × 10^3^ Pa, and the pressure under 9 m water depth is about 0.088 MPa. How do deep submergence environments affect the biosynthesis of the endogenous hormone GAs and thus inhibit plant growth?

For living cells, GAs promotes hydrolysis of hemicellulose by Xyloglucan Endotransfer glycosidase thereby softening and relaxing the cell wall and promoting cell elongation and cell division (Ayano et al., 2014; Wang and Komatsu, 2022). The percentage of S-phase cells significantly increased within 4~7 h of treating rice with GA_3_ through [^3^H] thymidine and DNA admixture experiments, suggesting that GA promotes cell division in meristematic tissues of internode and shortens the cell cycle, and that internode elongation in deep-water rice is ultimately regulated by GA (Fukao et al., 2019). Our findings are also consistent with previous studies that the response of plants in submerged environments is very closely linked to the GAs. Consequently, GAs content at the base of immature internodes of *A. philoxeroides* decreased with increasing water depth, and as we expected.

It was demonstrated in previous studies that mechanical stress has important effects on the biosynthesis of endogenous plant hormones (Chehab et al., 2012; Lange and Lange, 2015), such as the effect of soil stress on ethylene synthesis (Potocka and Szymanowska-Pułka, 2018). From a mechanical perspective, when the force environment is altered, the distribution of mechanical stresses within plant tissues also undergoes localized changes, followed by cascading effects at the cellular (Richter et al., 2009) and molecular (Laskowski et al., 2008) levels. Therefore, it is possible that the higher hydrostatic pressure under 9 m water depth resulted in changes in the distribution of mechanical stress in the immature internodes of *A. philoxeroides*, which in turn affected the biosynthesis of GAs. Hydrostatic pressure is a specific type of mechanical stress, and its effects on plant growth include the effects of submergence and the effects of force. The stress response produced by plants varies depending on the severity and duration of the stress occurrence (Shi et al., 2016). The growth response of plants should be different for different intensities of hydrostatic pressure and different times of action on the plants.

## 5 Conclusion

Entirely consistent with the conjecture, our results suggested that the stem elongation of *A. philoxeroides* responded significantly to submergence depth and duration, especially the response of immature stems. Elongation of immature stems decreased when water depth increased, which was associated with elongation at the basal of immature internodes, and the basal parts made the biggest contribution to the elongation of internodes. Moreover, the elongation of the basal part of the immature stems was related to the concentration of endogenous hormone GAs. Therefore, in flood-prone environments, *A. philoxeroides* was able to grow rapidly through clonal integration, and its growth response differences to water depth were regulated by endogenous hormones. The results of this study provide strong evidence to demonstrate the important role of the hydrostatic pressure induced by flooding. Investigating the growth response of plants in different water depth can deepen our understanding of clonal plant submergence tolerance mechanisms, and help to explore the future management of water level regulation in the drawdown zone of large reservoirs (e.g., Three Gorges Reservoir).

## Data availability statement

The raw data supporting the conclusions of this article will be made available by the authors, without undue reservation.

## Author contributions

SJ: Data curation, Investigation, Writing – original draft. XR: Data curation, Investigation, Writing – original draft. FL: Writing – review & editing. HN: Data curation, Writing – original draft. QA: Formal analysis, Methodology, Writing – review & editing. BW: Data curation, Writing – original draft. BZ: Conceptualization, Funding acquisition, Methodology, Writing – original draft. XZ: Conceptualization, Funding acquisition, Methodology, Writing – original draft, Writing – review & editing.
