# Supplementary material for: Stem elongation and gibberellin response to submergence depth in clonal plant Alternanthera philoxeroides
Source: Front Plant Sci. 2024 May 24;15:1348080. doi: 10.3389/fpls.2024.1348080 (PMC11157100; doi:10.3389/fpls.2024.1348080)
Supplement: Supplementary file 1 [file DataSheet_1.docx]

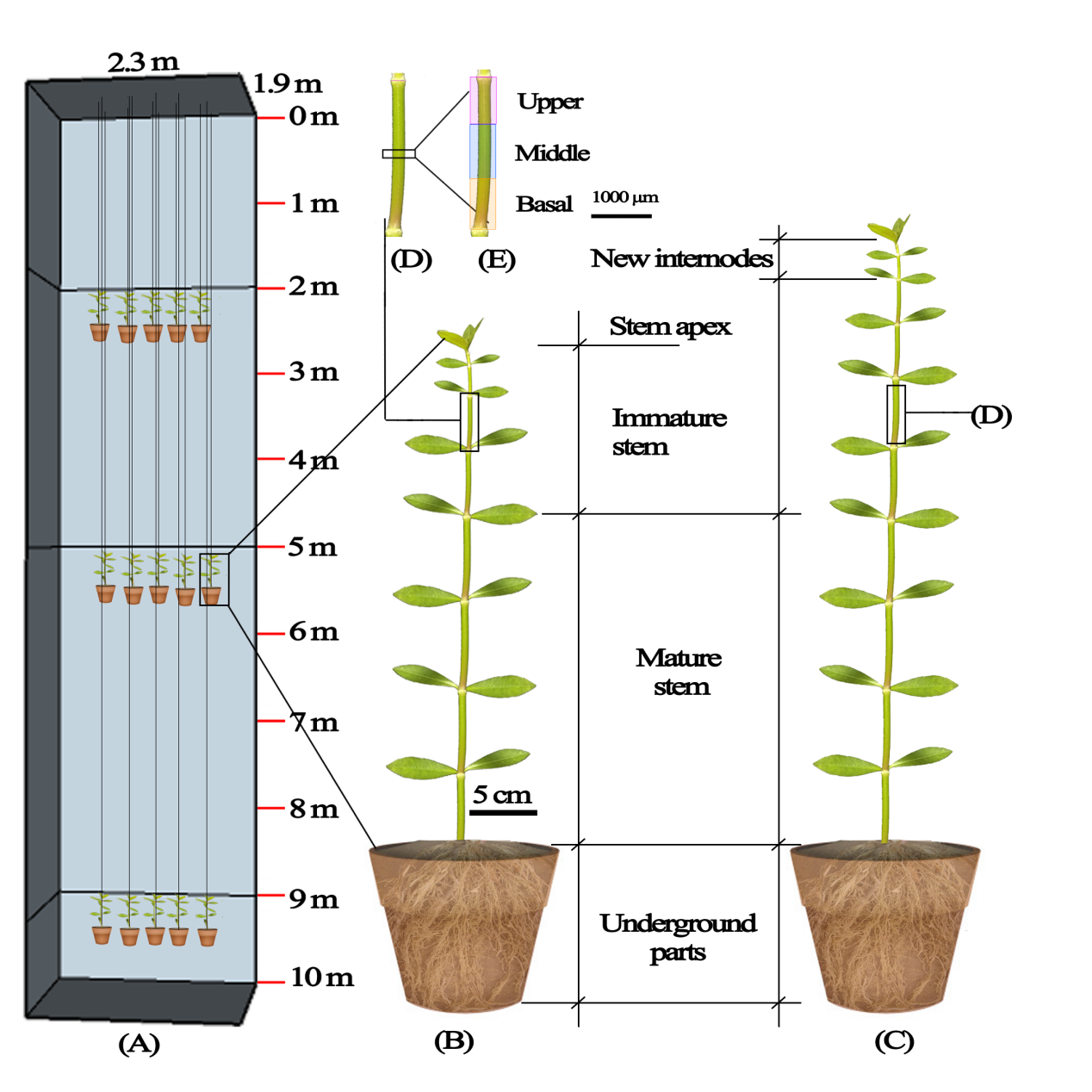
**Figure S1** Diagrammatic representation of the submergence design and plant material. (A) The frame of the 10 m deep concrete reservoir (length × width × depth= 2.3 m × 1.9 m × 10 m) and the 2 m, 5 m and 9 m submergence treatments; (B) underground parts, mature stem, immature stem and stem apex of *A. philoxeroides* before submergence treatments; (C) underground parts, mature stem, immature stem and new internodes, and an internode section of *A. philoxeroides* after submergence; (D,E) the basal, middle and upper part of an immature internode, marked before treatments and used for measuring daily growth at different depths of submergence.
